# The effect of internet embeddedness on women’s non-farm employment under the power perception perspective: evidence from rural China

**DOI:** 10.3389/fpsyg.2023.1248858

**Published:** 2024-01-24

**Authors:** Song Yu, Lulu Yang, Shimei Yang, Lianjun Li

**Affiliations:** ^1^Nanchang Business College of Jiangxi Agricultural University, Jiujiang, Jiangxi, China; ^2^College of Economic Management, Henan Agricultural University, Zhenzhou, Henan, China; ^3^College of Economic Management, Jiangxi Agricultural University, Nanchang, Jiangxi, China

**Keywords:** internet embeddedness, perception of rights, off-farm employment of women, women’s empowerment, gender equality

## Abstract

**Introduction:**

Female empowerment can promote gender equality and realize women’s comprehensive development, while the Internet has opened up an effective channel for female empowerment.

**Methods:**

Given the relative lack of women’s rights in rural China and the rapid Internet development, this paper, based on the 2021 China Comprehensive Social Survey data, first analyses the effect of Internet embeddedness on rural women’s non-farm employment using the Probit model. Then, it uses the mediation effect model to explore the role of women’s rights perception in the mechanism between Internet embeddedness and women’s non-farm employment. Finally, based on age and regional differences, we also analyze the impact of Internet embeddedness on female non-farm employment.

**Results:**

The paper draws the following conclusions: (1) Internet embedding can promote female non-farm employment, and the probability of female non-farm employment is 3.71% for each degree of Internet embedding. (2) Internet embedding can enhance women’s perception of their rights and thus promote women’s non-farm employment. (3) Internet embedding can enhance the perception of rights of young rural women, which can promote their nonfarm employment. However, the influence of internet embedding on middle-aged women’s perception of rights is not significant. (4) Internet embedding in the eastern region can enhance women’s perception of rights and realize women’s non-farm employment; on the contrary, in the central and western regions, Internet embedding cannot enhance rural women’s perception of rights.

**Discussion:**

Therefore, this paper proposes to release further the impact of the Internet in promoting rural women’s non-farm employment to improve the deprivation of rural women’s rights and promote gender equality and women’s comprehensive development.

## 1 Introduction

Female empowerment is an effective way to promote gender equality and achieve the comprehensive development of women, which is of great significance in promoting the progress of social civilization. For a long time, women, as a relatively disadvantaged group in rural areas (Li et al., 2022), women enjoy limited autonomy in the household in rural China, and they have less say than men in the disposition of household resources and decision-making in matters (Tang and Luo, 2014). Therefore, in the face of the increasing contradiction between women’s willingness to meet their needs for a better life and the imbalance between women’s underdevelopment, rural women urgently need to change their lack of rights, and the call for women’s empowerment continues to rise.

With the popularization and application of information and communication technology in recent years (Wei et al., 2023), the internet has developed rapidly in China. According to data from the China Internet Development Statistics Report, as of June 2022, the total number of Chinese internet users reached 1.051 billion, and the internet penetration rate reached 74.4%; among them, rural areas accounted for 27.9%, and women accounted for 48.3%. According to the China Internet Development Statistics Report, as of June 2022, the total number of Chinese internet users reached 1.051 billion, and the internet penetration rate reached 74.4%. Among them, the number of people in rural areas accounted for 27.9%, and the number of women accounted for 48.3%. As a major breakthrough of the third technological revolution, the Internet profoundly affects efficiency and equity (Zhang et al., 2019). The digital revolution has changed how individuals work and live (Ghobakhloo, 2020). The rise of various “network chicken soup text” and “network feminist consciousness” is constantly reshaping rural women’s awareness of their rights (Chen and Gui, 2023). At the same time, with the rapid development of the digital economy and the deepening of urbanization, women have gained economic independence by participating in non-agricultural employment in the market, which has increased their voice in the family (Chen, 2020). The fact that rural women in China improve their family status through non-agricultural employment has gradually become a generally recognized fact in society (Li and Feng, 2022).

A great deal of research is now being conducted in the academic community on the nonfarm employment of rural women. The following conclusions were drawn regarding the analysis of factors affecting female nonfarm employment. Poverty alleviation relocation (Zhu et al., 2022) and farmland transfer (Cai et al., 2018) can significantly promote female nonfarm employment. Among them, farmland transfer increases rural women’s duration of local nonfarm employment, while the effect of duration of outbound nonfarm employment is not significant. Additionally, female nonfarm employment decisions depend on nonfarm employment income compared to the cost of child care, and intergenerational care significantly contributes to rural young women’s nonfarm employment by reducing the cost of child care (Hua and Chen, 2018). The analysis of the benefits of female nonfarm employment has the following conclusions. Nonfarm employment reduces female farming activity and strengthens female empowerment (Maligalig et al., 2019). Off-farm employment also increases female earnings, which helps to improve the living standards of households and thus enhances women’s subjective well-being (Van den Broeck and Maertens, 2017). At the same time, the increase in female earnings can motivate women to invest in their health and help improve their physical and mental health (Shen et al., 2022). The following studies have been conducted to analyse female nonfarm employment constraints. The burden of sick care can discourage female nonfarm employment (Wang et al., 2020). In addition, the constraints on nonfarm employment for rural married women will deepen due to traditional Chinese family division of labor patterns, child care and elderly support (Zhou, 2013). Moreover, health insurance can reduce female off-farm employment, especially for farm households with predominantly smallholder business practices (Liao and Taylor, 2010). The distance between the farm household and the city is also an important constraint on female nonfarm employment. Female nonfarm employment is limited to different degrees depending on the distance from the city (De Janvry and Sadoulet, 2001). The analysis of the form of female nonfarm employment has the following conclusions. Women have a lower share of nonfarm employment overall than men, but women are more likely to be in permanent nonfarm employment (Xie et al., 2022).

In conclusion, the previous academic exploration has accumulated a strong theoretical foundation and practical experience for the analysis of this paper, but there are still areas worth exploring. Previous scholars have mostly explored the inner logic of women’s nonfarm employment from the material level, such as farmland transfer, child care costs, and elderly support costs, while neglecting the analysis of the spiritual level, such as women’s rights perception. However, the stronger the perception of women’s rights and the louder the call for women’s empowerment, the stronger the endogenous motivation for women to acquire family status through non-agricultural employment. Therefore, it is necessary to explore the internal logic of women’s non-agricultural employment from the perspective of women’s rights perception, which is more in line with current social development. In addition, how does the rapid development of the Internet by the third technological revolution affect women’s perception of their rights? Will the lack of rural women’s rights be further aggravated by technological barriers or the information gap? Or will the Internet’s restructuring of rural women’s discourse further enhance their perception of their rights? Given the above discussion, this paper explores the impact of Internet embeddedness on women’s non-agricultural employment based on women’s perception of rights. The research idea of this paper includes the following three aspects: (1) Using the Probit model to analyze the impact of Internet embedding on female non-farm employment. Since female non-farm employment is a type II variable, this paper adopts the Probit model, which can ensure the accuracy of the research results. (2) Using the mediation effect model to explore the role of women’s rights perception mechanism. Due to a certain degree of distortion in the stepwise test method, this paper adopts the Sobel and Bootstrap tests to analyze the results. (3) Based on age and regional differences, this paper explores the effect of Internet embeddedness on female non-farm employment and the mediating effect of female rights perception.

The marginal contributions of this paper are the following two: First, this paper analyzes the impact of Internet embeddedness on female non-agricultural employment from the perspective of rights perception, which is conducive to combining women’s demands for rights with actual social development to explore effective ways to achieve gender equality. Second, based on age and regional differences, this paper discusses the differences and factors of the impact of Internet embeddedness on rural women’s perception of rights and then affects female non-agricultural employment, which is helpful to provide decision-making ideas for macro policy-making in China and even other regions of the world.

## 2 Theoretical analysis and research hypotheses

### 2.1 Internet embedding and women’s nonfarm employment

A review of the previous literature reveals that the effect of the internet on female nonfarm employment is mainly reflected in the following aspects. First, the internet increases rural women’s off-farm labor hours. The internet has the advantages of timely and convenient information delivery (Zhuo and Lu, 2020), which enables rural women to have quick access to various information. Access to relevant information enables rural women to improve their way of completing their affairs and agricultural production, which leads to the improvement of the efficiency of domestic work and agricultural production (Vazquez and Winkler, 2017). As a result, rural women’s off-farm work time has increased. Second, the internet can increase the accumulation of human capital and social capital among rural women. The internet can provide efficient and low-cost information, which transmits knowledge and skills for nonfarm employment to rural women and promotes their own human capital accumulation (Pan et al., 2021). The internet also broadens the social scope of rural women by enhancing the ease of interpersonal interaction and communication, which increases the social capital accumulation of rural women (Peng et al., 2022). Third, the internet has provided a large number of employment opportunities for rural female nonfarm transfers. With the development of the internet platform economy, new industrial forms and new forms of employment represented by online platform-based employment have emerged in large numbers (Mo and Li, 2022); for example, the sharing economy based on the application of digital technology has facilitated the development of the “odd-job economy” (Tian and Zhang, 2022). Moreover, the development of the digital economy has given rise to the consumer internet, which has stimulated the industry’s demand for low-skilled labor, such as couriers and delivery workers (Liu and Li, 2017). In addition, networked consumption and transactions have greatly increased the flexibility of work, which provides more liberal work opportunities for low-skilled workers (Foucault, 1970). In conclusion, with the popularization and development of the internet, rural women’s nonagricultural time has been expanded, and their market competitiveness has been enhanced. At the same time, the internet has given rise to new industries and new forms of employment, which eventually prompted rural women to shift to nonagricultural industries.

Based on the above analysis, this paper proposes the following research hypothesis.

*H1*: Internet embedding can promote female nonfarm employment.

### 2.2 Internet embedding and rights perception

The article analyses rural women’s rights perceptions based on Foucault’s discourse theory. Foucault incorporates elements such as knowledge and discourse into the framework of the connotative analysis of rights, arguing that rights are bred in the creation of identity and discourse (Xiong, 2021). Discourse rights refer to the ability to use discourse as a guide to control social opinion and ideas, which directly or indirectly influences others’ ideological judgments and action orientations (Qi et al., 2022).

For a long time, the economic space for rural women has been extremely squeezed by both the external demand of the labor market and traditional ideology (Liu, 2018), which has led to the economic status of rural women being subordinate to men. This has resulted in the deprivation of their rights. The lack of discourse rights of rural women is extremely serious. The popularity and development of the internet has provided channels and opportunities for rural women to express their demands, and the internet has the advantages of convenience, timeliness, and the powerful dynamics of online public opinion. It eventually led to the reshaping of rural women’s discourse (Ye and Xu, 2019). First, the internet has decentralized discourse rights. The multinode and centre-less design of the internet has weakened the traditional unidirectional power structure, which has contributed to the fragmentation and diffusion of rights (Yan, 2021), ultimately making it possible for rural women to regain their discursive rights. In addition, the information and communication technology of the internet is deeply embedded in network discourse rights, which makes network discourse rights open, decentralized and mass. The network discourse right makes rural women break the time and space interval and the right regulation to transmit information and exchange ideas. This leads to the formation of a two-way interaction of information and a pluralistic and diffuse network pattern (Liu, 2021). Second, the internet is driving the transformation of rural women’s messaging identity. The technological features of the internet have technologically empowered the expression of women’s discourse rights, which enables rural women to exercise their discourse rights conveniently. The technological empowerment of rural women’s discourse rights by the internet has led to the transformation of women from a single demand for information resources to a dual identity of information demander and information supplier (Yu et al., 2015), which has led to the further enhancement of their discourse rights. Therefore, in the internet era, discourse rights show decentering characteristics. The relatively powerless groups in society achieve mutual support and cohesion through the internet (Liu, 2022), which invariably enhances their discourse rights. Faced with the reality of economic development and technological progress, the regaining of women’s discourse rights can stimulate their rights awareness, which makes them more concerned about their rights enjoyment and rights deficit. The increased awareness of rural women’s power motivates them to achieve their rights through practical actions (Haidt and Rodin, 1999). It has been shown that individuals’ perceptions of entitlement have a greater impact on behavior than the actual entitlement they have (Wen et al., 2005). As women’s perceptions of their rights increase, they become increasingly aware of their own economically passive position. At the same time, the development of the internet has significantly compensated for the previous shortcomings of rural women in terms of knowledge skills and access to information. This has resulted in rural women choosing nonfarm employment to improve their economic disadvantage.

Based on the above analysis, this paper proposes the following research hypothesis.

*H2*: Perceived rights can strengthen the impact of internet embedding on rural women’s nonfarm employment. Internet embedding can increase women’s perception of rights, which promotes rural women’s nonfarm employment.

## 3 Data and methods

### 3.1 Data sources

The data in this paper are obtained from the Chinese General Social Survey (CGSS) database, and the 2021 panel data in the database are selected as the basis of the study. The data cleaning process in this paper is as follows. First, we select the sample variables of the paper. Since the article analyses the effect of the internet on female nonfarm employment, it focuses on the selection of variables related to farmers’ personal characteristics, household characteristics, and the internet and removes irrelevant variables to retain a total of 13 variables. Second, the sample data, such as extreme values, abnormal values, and missing values, are processed. Finally, 2037 data points were obtained through a series of data processing processes.

### 3.2 Variable

(1) The dependent variable in this study is the nonfarm employment of women. The questionnaire was based on the question “Respondents’ current work status.” If the respondent works in agriculture, the assignment is 0. If the respondent works in a nonagricultural job, the assignment is 1 (See Table 1).
(2) The core variable in this study is internet embedding. Since this paper explores the impact of internet embedding on women’s nonfarm employment and focuses on women’s ability to use the internet to obtain effective information, the degree of women’s internet embedding is selected as the core variable in this paper. The questionnaire was based on the question “Whether the respondent’s information mainly comes from the internet (including mobile internet).” If the answer is “Yes,” the assignment is 1; otherwise, the assignment is 0 (See Table 1).
(3) The mediating variable in this study is women’s perception of rights. Referring to Foucault’s theory of discourse rights and combining the analysis of the former literature, this paper divides women’s rights perception into three levels: identity perception, competence perception, and self-identity. The questionnaires were based on the questions “Do you agree with the following statement: men put their careers first and women put their families first?,” “Do you agree with the following statement: men are naturally more capable than women?,” “Do you agree with the following statement: it is better to do well than to marry well?.” Subsequently, the model of actor analysis was used to reduce the dimensionality of these three types of questions to obtain the mediating variables characterizing women’s rights perceptions (See Table 1).
(4) Referring to the relevant literature, the article also controls for other variables affecting female nonfarm employment, such as demographic variables, household characteristics variables, and individual characteristics variables. Among them, demographic variables include female age; household characteristics variables refer to household size and region; personal characteristics variables refer to educational status, health status, marital status, and election status (See Table 1).
(5) The instrumental variable in this study is women’s frequency of internet access. This article explores the impact of internet embedding on women’s nonfarm employment, focusing on women’s ability to use the information channels of the internet to obtain effective information. However, there is no direct link between women’s frequency of internet access and their nonfarm employment. Meanwhile, women’s frequency of internet access is highly correlated with women’s degree of internet embedding. This suggests that women’s frequency of internet access is consistent with the correlational, exogenous characteristics of the instrumental variable (See Table 1).

**Table 1:** Descriptive statistical analysis.

Variable type	Variable name	Definition	Mean	SD ^a^	Min	Max
Dependent variable	Nonfarm employment of women	Respondents’ current work status ^b^	0.42	0.49	0	1
Core variable	Internet embedding	Respondents’ information mainly coming from the internet (including cell phone access) (No = 0; Yes = 1)	0.55	0.5	0	1
Mediating variable	Women’s perception of rights	This variable was calculated by factor analysis and it is a continuous variable	2.7	1	−1.96	1.97
Control variables	Age	Respondent age, which is a continuous variable	39.9	9.92	17	54
	Educational status	Highest education level of respondents ^c^	2.2	0.76	1	4
	Health status	Respondent’s current health status ^d^	3.67	1.07	1	5
	Election status	Whether the respondent voted in the last village council election (No = 0; Yes = 1)	0.41	0.49	0	1
	Household size	Number of respondents’ households living together, which is a continuous variable	3.33	1.48	1	11
	Marital status	Respondent’s current marital status ^e^	0.87	0.33	0	1
	Region	Region where the respondent is located ^f^	2.04	0.78	1	3
instrumental variable	Frequency of internet access	In the past year, did the respondent often engage in internet activities during their free time ^g^	3.58	1.75	1	5

^a^SD, Standard deviation; ^b^ 0, agricultural, 1, nonagricultural; ^c^ 1, illiterate, 2, elementary and junior high school, 3, high school and junior college, 4, college and above; ^d^ 1, very unhealthy, 2, relatively unhealthy, 3, average, 4, relatively healthy, 5, very healthy; ^e^ 0, no spouse, 1, with spouse; ^f^ 1, West, 2, Central, 3, East; ^g^ 1, never; 2, several times a year or less; 3, several times a month; 4, several times a week; 5, every day.

### 3.3 Empirical method

#### 3.3.1 Binary probit model

This study focuses on identifying the impact of internet embedding on rural female nonfarm employment. The dependent variables are whether rural women are engaged in nonfarm employment, engaged in agricultural employment Y*_i_* = 0, and engaged in nonfarm employment Y*_i_* = 1, which are dichotomous variables. Therefore, the study developed the following binary probit model:

(1) $$Y_{i}=\alpha +\mathrm{\beta Interne}t_{i}+\gamma X+\varepsilon_{i}$$

Internet*_i_* denotes the degree of internet embedding, X denotes the vector of control variables, α, β, γ denotes the parameters, and εi denotes the random interference term.

#### 3.3.2 Intermediary effect model

Referring to the research of Wen et al. (2005), this study uses an intermediary effect model to test the mechanism of action between internet embedding and rural female nonfarm employment. Therefore, the study developed the following binary mediating effects model.

(2) $$Y=Z_{1}+\mathrm{aInterne}t_{i}+b_{1}X_{1}+\theta_{1}$$

(3) $$M=Z_{2}+\mathrm{cInterne}t_{i}+b_{2}X_{2}+\theta_{2}$$

(4) $$Y=Z_{3}+\mathrm{dInterne}t_{i}+eM+b_{3}X_{3}+\theta_{3}$$

Y denotes rural female nonfarm employment, interneti denotes the degree of internet embedding, M denotes women’s perception of rights, X denotes control variables, Z denotes constant terms, and θ denotes the random interference term. (2) denotes the total effect of internet embedding on rural female nonfarm employment, and (3) denotes the effect of internet embedding on women’s perception of rights. The coefficient e in (4) denotes the direct effect of women’s perception of rights on rural female nonfarm employment. Substituting (3) into (4) yields the indirect effect of rural female nonfarm employment ec, which is the effect of internet embedding on rural female nonfarm employment by enhancing women’s perception of rights. This paper uses the logit model to analyse the equations.

## 4 Empirical analysis

### 4.1 The benchmark regression analysis

Table 2 reports the analysis of the benchmark regression results of internet embedding on rural female nonfarm employment^1^. Model 1 is a correlation between rural female nonfarm employment and internet embedding. Model 2 estimates a regression with both internet embedding and women’s perception of rights in the model, and Model 3 is a further addition of control variables to Model 2. The estimated coefficients of internet embedding are positively significant in several models. This indicates that internet embedding promotes rural female nonfarm employment, and the results are somewhat robust.

**Table 2:** Results of the baseline regression analysis.

Variable	(1)	(2)	(3)
Internet embedding	0.786^***^ (13.45)	0.645^***^ (10.58)	0.371^***^ (4.92)
Nonfarm employment of women		0.284^***^ (9.23)	0.209^***^ (6.22)
Age			0.004 (0.86)
Educational status			0.308^***^ (6.18)
Health status			0.172^***^ (5.40)
Election status			−0.261^***^ (−4.05)
Household size			−0.011 (−1.06)
Marital status			−0.382^***^ (−3.77)
Region			0.302^***^ (7.58)
_cons	−0.648^***^ (−14.43)	−0.577^***^ (−12.53)	−2.024^***^ (−6.92)
*Log likelihood*	−1294.5426	−1250.8924	−1118.2785
*Pseudo R2*	0.067	0.0984	0.1762
*N*	2037		

*t* statistics in parentheses ***p < 0.001.

As shown in Model 3, internet embedding significantly and positively influences rural female nonfarm employment. Specifically, with other conditions unchanged, for each unit increase in internet embedding, the probability of rural female nonfarm employment increases by 37.1%. The reasons for this result are as follows: (1) The deeper the Internet is embedded, the lower the opportunity cost for women to search for information on non-agricultural employment. In the face of the reality that there are many family affairs, women can maximize their access to effective information at the lowest cost through the Internet, alleviating the psychological pressure arising from non-agricultural employment. (2) The more deeply embedded the Internet is, the more women can use it to optimize their human and social capital, thus improving their market competitiveness and probability of employment.

In addition, the results also show that: (1) The younger the age, the more significant the tendency of rural women to be employed in non-agricultural employment. Young rural women have more familiar social relations in rural areas and can collect relevant information sensitively, increasing the probability of non-farm employment (Su and Zhou, 2005). (2) The higher the education level, the more significant the tendency of rural women to be employed non-farm. The higher the education level, the higher the women’s expectation of future earnings, and the stronger the education level, the stronger the women’s self-adaptation ability is (Liu and Yu, 2022). Therefore, in the face of lower marginal returns from farming and higher marginal returns from labor, the non-farm employment tendency of women with higher education is more obvious. (3) The better the health status, the more significant the non-farm employment tendency of rural women. Health, as an important module of human capital, is significant in improving individual human capital accumulation (Guo and Wei, 2020). The better the health condition of rural women, their human capital tends to be optimized, which leads to more advantages in choosing a job, so they show a stronger propensity for non-farm employment. (4) The rural women who lack the right to vote, the more significant their non-farm employment tendency. Electoral rights are an important means for rural women to participate in village governance, which, to a certain extent, can reflect the economic independence of women (Yang et al., 2023). The lack of village election rights reflects women’s lower participation in village governance, lack of economic independence and lower sense of belonging to the village. Therefore, women favor non-farm employment to change the status quo. (5) Married rural women have a lower tendency to engage in non-farm employment. Married women must consider many factors, such as the cost of caring for young children and supporting older people. At the same time, due to the limited social network relationship and labor supply ability (Li and Wang, 2020), the opportunity cost of married women engaged in non-agricultural employment is relatively large. (6) The closer rural women are to the eastern region, the more significant their non-farm employment tendency. The geographical location, infrastructure, education level, and economic development level of the eastern region are significantly stronger than those of the central and western regions (Wang and Zhang, 2022), which makes the marginal returns to female labor significantly higher than the marginal returns to farming. Therefore, rural women near the eastern region have a greater tendency to choose non-farm employment.

### 4.2 Intermediary effect analysis

According to the theoretical analysis above, the popularization and development of the Internet provide channels and opportunities for rural women to express their demands, reshape their discourse power, and enhance women’s awareness of rights. To change their long-term weak economic position, women choose non-agricultural employment through the skills training and employment information provided by the Internet to increase economic income. Therefore, referring to the research of Wen et al. (2005), this paper uses the intermediary effect model to test the impact of women’s perception of rights on the relationship between network embeddedness and rural women’s non-agricultural employment.

First, this paper uses a stepwise approach to analyze the effect of women’s perceived rights on the relationship between network embeddedness and rural women’s non-farm employment^2^. Table 3 reports the test results of the stepwise method. Model (2) shows that Internet embeddedness can enhance women’s perception of rights. Compared with Model (3) and Model (2), the influence coefficient of Internet embeddedness on female non-agricultural employment has decreased, indicating that female rights perception partially mediates between Internet embeddedness and rural female non-agricultural engagement.

**Table 3:** Stepwise approach analysis of results.

Variable	(1)		(2)		(3)	
	Nonfarm employment of women		Women’s perception of rights		Nonfarm employment of women	
	Coefficient	Standard error	Coefficient	Standard error	Coefficient	Standard error
Internet embedding	0.410^***^	5.51	0.225^***^	3.99	0.371^***^	4.92
Women’s perception of rights	–	–	–	–	0.209^***^	6.22
Control variables	Controlled		Controlled		Controlled	
Log likelihood	−1137.8316		−7655.5105		−1118.2785	
Pseudo R2	0.1618		0.0223		0.1762	
*N*	2034					

*t* statistics in parentheses ***p < 0.001.

Second, the results are analyzed again in this paper using the Sobel test and Bootstrap test to ensure the robustness of the results. Table 4 reports the results of the intermediary effect of input records. The results showed that the indirect effect passed the significance test of 5%, the coefficient was positive, and the indirect effect accounted for 9.87%, indicating that women’s perception of rights played a partial mediating effect between internet embedding and rural female nonfarm employment. Internet embedding can enhance rural women’s perception of rights, which promotes nonfarm employment. This verifies research Hypothesis H2. The reason for this result is that the Internet has reconfigured rural women’s discourse through information and communication technologies: (1) Rural women have gained more channels to express their demands for rights, making them more aware of their lack of rights and thus showing stronger demands for rights, especially regarding economic rights. (2) Women are more emotionally rich and sympathetic, thus more likely to form a rights defense group online. Once a group member is treated unfairly, other team members will act collectively to try to change the individual’s status quo, thus demonstrating stronger synergy. Therefore, the more deeply embedded the Internet is, the stronger rural women’s awareness of their rights is, and their tendency to take up non-agricultural employment on the advice of group members is more significant.

**Table 4:** Intermediary effects of input records.

Action mechanism	Indirect effects as a percentage (%)	Intermediary effects	95% confidence interval		*Z* value
			Upper limit	Lower limit	
Internet embedding→Women’s perception of rights→Nonfarm employment of women	9.87	0.014^**^ (3.29)	0.006	0.023	3.29

*t* statistics in parentheses **p < 0.01.

### 4.3 Heterogeneity analysis

#### 4.3.1 Age heterogeneity

There may be age differences in the impact of internet embedding on rural women’s nonfarm employment. Therefore, this paper divides rural women in the sample into young women and middle-aged women according to their ages. Among them, young women refer to rural women aged 17–45, and middle-aged women refer to rural women aged 46–55 to further verify the age difference in the impact of internet embedding on rural women’s nonagricultural employment.

Table 5 reports the age difference analysis of the impact of internet embedding on rural women’s nonfarm employment. The results show that internet embedding can enhance the perception of rights of young rural women, which can promote their nonfarm employment. However, the influence of internet embedding on middle-aged women’s perception of rights is not significant. This result is because young women are more capable of familiarizing themselves with new things and are more eager to seek economic independence. Therefore, Internet embeddedness can effectively stimulate their awareness of their rights and, at the same time, understand their lack of ownership so that Internet embeddedness is more likely to promote their choice of non-agricultural employment. On the contrary, being older, they need to consider more things, such as the age limit for hiring, the education limit (older rural women in China generally have lower education levels), child care, etc., which seriously constrain their tendency to choose non-agricultural employment, even if the Internet does enhance their awareness of their rights.

**Table 5:** Age heterogeneity.

Region	Intermediary effects	95% confidence interval	*p* value
Youth	0.02^***^ (3.39)	[0.00950.0325]	0.001
Middle Age	0.006 (0.94)	[−0.00570.0180]	0.350

*t* statistics in parentheses ***p < 0.001.

#### 4.3.2 Regional heterogeneity

There may be regional differences in the impact of internet embedding on rural female nonfarm employment. Therefore, this paper further verifies the regional differences in the impact of internet embedding on rural female nonfarm employment by dividing rural women’s locations into three regions: eastern, middle, and western.

Table 6 reports the regional difference analysis of the impact of internet embedding on rural female nonfarm employment. The results show that internet embedding enhances women’s perceptions of rights in rural eastern China, which leads them to engage in nonfarm employment. However, for rural women in the middle and western regions, internet embedding does not enhance their perception of rights. The eastern region has complete infrastructure and high-quality Internet talents for Internet development, which makes the Internet development environment better and the development level higher. Therefore, the region has a strong capacity for technological innovation. As a result, women in the eastern rural areas have a higher degree of Internet access and a stronger sense of safeguarding their rights. Moreover, many new business forms and forms relying on the Internet platform economy have emerged in the region, and the labor market demand is strong. In addition, women in eastern rural areas can rely on the advantages of geographical location; on the one hand, they can achieve nearby employment and relieve the psychological burden of being far from home. On the other hand, it can achieve flexible employment and reasonably arrange non-farm employment time and family affairs time. Therefore, women in the eastern rural areas have a more significant propensity to non-farm employment than those in the central and western regions.

**Table 6:** Regional heterogeneity.

Region	Intermediary effects	95% confidence interval	*p* value
East	0.024^**^ (2.64)	[0.008, 0.044]	0.008
Middle	0.009 (1.27)	[−0.004, 0.025]	0.205
West	0.008 (1.37)	[−0.001, 0.022]	0.170

*t* statistics in parentheses **p < 0.01.

### 4.4 Heterogeneity analysis

#### 4.4.1 Substitution of variables

There may be a certain lag in the impact effect of Internet embedding on rural female non-farm employment, thus affecting the accuracy of the research results. Since the latest database data used in this paper is data from 2021, this paper takes the data from 2017 as the baseline to analyze the impact of Internet embeddedness on rural women’s non-farm employment. At the same time, this paper adopts the method of replacing the dependent variable to examine the effect of Internet embedding on the non-farm jobs of rural women in 2018 and 2021, respectively.

Table 7 reports the results of the study. The results show that internet embedding promotes rural women to take up non-farm employment not only in the current period but also in 2018. This further validates the robustness of this paper’s findings. However, in 2021, Internet embeddedness does not promote female nonfarm employment, which suggests that there is some time limit to the impact effect of Internet embeddedness on female nonfarm work.

**Table 7:** Substitution of variables analysis results.

Variable	(1)		(2)		(3)	
	2017		2018		2021	
	Coefficient	Standard error	Coefficient	Standard error	Coefficient	Standard error
Internet embedding	1.229^***^	20.05	0.153^**^	2.78	0.0171	0.36
Women’s perception of rights	0.262^***^	6.75	——	——	——	——
Control variables	Controlled		Controlled		Controlled	
Log likelihood	−1160.3616		−1476.1336		−1932.8158	
Pseudo R2	0.1994		0.0026		0.0000	
*N*	2,179					

*t* statistics in parentheses **p < 0.01, ***p < 0.001.

#### 4.4.2 Influence effect measurement

To verify the accuracy of the study findings, this paper uses propensity score matching to measure the average treatment effect of internet embedding on rural female nonfarm employment. The article divides internet embedding into an experimental group (network embedded) and a control group (network unembedded) and uses nearest-neighbor matching and kernel matching to measure the average treatment effect of internet embedding on rural women’s nonfarm employment. Then, the average of the average treatment effects of the two groups measured by nearest-neighbor matching and kernel matching is taken to estimate the effect of internet embedding on rural women’s nonfarm employment. Due to space limitations^3^, this paper only presents the measured effects of internet embedding on rural female nonfarm employment.

Table 8 reports the results of the impact effect measurements. The results show that internet embedding can significantly promote rural female nonfarm employment with an impact effect of 0.164, i.e., for each unit increase in internet embedding, the probability of rural female nonfarm employment increases by 16.4%. This is consistent with the test results of the baseline regression, which indicates that the regression results are robust.

**Table 8:** Impact efficiency measurement results.

Matching method	ATT	Standard error	*T* test value
Nearest-neighbour matching	0.247^***^	0.024	10.49
Kernel matching	0.081^*^	0.034	2.41
Mean	0.164		

*t* statistics in parentheses *p < 0.05, ***p < 0.001.

#### 4.4.3 Instrumental variable method

Since the endogeneity problem arising from reverse causality may affect the estimation of the results, this paper selects internet access frequency as the instrumental variable and uses two-stage least squares (2SLS) to verify the accuracy of the baseline regression results. The instrumental variables selected for the article satisfy two requirements. On the one hand, the instrumental variables are consistent with correlation. The frequency of internet access is highly correlated with internet embedding. On the other hand, the instrumental variables are consistent with exogeneity. Women in rural areas may choose to engage in nonfarm employment only after broadening their horizons and enhancing their capabilities and perception of their rights through the various types of information provided by the internet. However, there is no direct relationship between the frequency of internet access by rural women and their nonfarm employment.

Table 9 reports the results of the 2SLS estimation. The results of the first stage showed that the higher the frequency of internet access, the deeper their internet embedding, which indicates a significant positive correlation between the frequency of internet access and the degree of internet embedding^4^. The second-stage regression results show that internet embedding passed the 1% significance test with a positive coefficient, i.e., the deeper the internet embedding, the higher the probability of rural women choosing to engage in nonfarm employment. For each unit increase in internet embedding, the probability of rural female nonfarm employment increases by 37.1%. This is consistent with the conclusions drawn in the previous paper and further tests the robustness of the findings in this paper.

**Table 9:** Two-stage least squares (2SLS) estimation results.

Variables	The first stage	The second stage
Internet embedding		0.371^***^ (9.47)
Frequency of internet access	0.174^***^ (36.39)	
*F*-statistic value	615.14	
*N*	2034	

*t* statistics in parentheses ***p < 0.001.

## 5 Discussion

This paper uses data from the 2021 Chinese General Social Survey to empirically analyse the correlation between internet embedding and rural female nonfarm employment and analyses the mechanism of the impact of internet embedding on rural female nonfarm employment. In contrast to previous literature, this paper analyses the mechanisms of internet embedding on rural women’s nonfarm employment from a rights-perception perspective.

In contrast to the literature, this paper finds that internet embedding reshapes rural women’s discursive rights and enhances women’s perceptions of rights, thereby facilitating rural women’s choice to engage in nonfarm employment. We also found that internet embedding enhances rural young women’s perceptions of rights. This indicates that young women are more aware of their rights, i.e., internet embedding can further increase their awareness of their perceived rights. At the same time, young rural women continue to empower themselves for nonfarm employment through various effective information and channels provided by the internet and eventually choose to engage in nonfarm employment. In addition, eastern China has a higher level of economic and social development than the middle and western regions, and the region exhibits higher industrial density, more flexible employment practices, more robust infrastructure, and more extensive social security services, which makes the propensity for nonfarm employment more pronounced for rural women in eastern China. In the case of internet embedding, the probability of nonfarm employment among rural women in eastern China is much higher than that in middle and western China.

There are some similarities and differences between the findings of this study and those of existing studies; specifically, internet use can promote rural female nonfarm employment. In line with Wang and Lu, research Hypothesis H1 is confirmed. Wang believes that the internet facilitates off-farm employment by changing the gender perceptions of rural women (Lu et al., 2023). Lu also believes that the development of the digital economy has contributed to the formation of the gender equality concept (Novo-Corti et al., 2014). Moreover, Novo-Corti believes that ICT can alleviate the digital divide for rural women and promote off-farm employment for rural women by increasing their awareness of gender equality (). The above study partially justifies research Hypothesis H2. All of the above emphasize the impact of the internet on rural women’s perceptions of gender equality, neglecting the analysis of women’s perceptions of their own occupational suitability and competence. Therefore, this study further analyses the impact of the internet on rural nonfarm employment and the analysis of the mechanism based on the abovementioned studies, with reference to Foucault’s discourse theory, and incorporates occupational fit and competence identity into the analytical framework.

This study has come up with the following ideas on the need to further unleash the impact effects of the internet in promoting rural women’s nonfarm employment and increasing women’s sense of perceived power. First, the government should strengthen the construction of rural network base stations to increase rural women’s network accessibility, which can help break the spatial and temporal constraints faced by rural women. Second, the government should create an online rights platform to provide solid conditions for rural women to express their rights, which will help expand the voice of rural women and enhance their awareness of their rights. Third, the government should develop new forms and businesses that help rural women’s nonfarm employment, such as live internet sales, the “odd job economy” and the “ground stall economy,” which can help expand new channels for rural women’s nonfarm employment.

In addition, there are certain shortcomings in this study. This study only examines the effect of the internet on rural female nonfarm employment in China, and the applicability of the findings to other regions needs to be further explored. In addition, this study constructs an analytical framework of occupational identity, competence identity and self-identity based on Foucault’s discourse theory and subsequently derives the values of indicators characterizing women’s perception of rights through a factor analysis method. This may not be comprehensive enough to draw conclusions.

## 6 Conclusion

Using data from the 2021 Chinese General Social Survey, this paper empirically analyses the effects, mechanisms of action, and heterogeneity of internet embedding on rural women’s nonfarm employment from the perspective of women’s rights perceptions. The article draws the following three conclusions.

(1) The Internet promotes rural women’s off-farm employment, but it is affected by rural women’s behavior and awareness. The Internet increases the probability of rural female non-agricultural employment by reducing the marginal cost of information supply. When rural women can fully use the advantages of low information acquisition cost brought by the Internet and consciously change their disadvantages of insufficient information acquisition ability, they can achieve non-agricultural employment. Therefore, the government or society must pay more attention to improving rural women’s abilities and provide various training opportunities to enhance women’s self-coping abilities.
(2) The Internet promotes the reconstruction of rural women’s discourse power, thus enhancing women’s perception of rights and constantly raising the voice of female empowerment. In the current reality that rural women’s economic rights are relatively lacking, realising non-agricultural employment through the Internet has become an effective way for rural women to regain their economic rights. Therefore, realising the joint development of the Internet and women’s empowerment is a practical problem that should be faced in realizing gender equality and promoting women’s all-round development.
(3) The level of Internet development profoundly impacts the degree of Internet embeddedness and non-agricultural employment of rural women. Rural women are more deeply embedded in the Internet in areas with a higher level of Internet development, so their awareness of rights protection is stronger, such as in eastern China. At the same time, areas with higher levels of Internet development have stronger technological innovation capabilities, thus providing rural women with various flexible employment methods.

## Data availability statement

The original contributions presented in the study are included in the article/supplementary material, further inquiries can be directed to the corresponding author.

## Author contributions

LL: conceptualization and writing—review and editing. SoY: methodology, investigation, and writing—original draft preparation. SoY and LY: formal analysis. ShY: data curation and supervision. All authors have read and agreed to the published version of the manuscript.

## Footnotes

^1^The primary test here is Equation 1, which examines the impact of Internet embeddedness on rural women’s nonfarm employment.

^2^The primary tests here are Equations 2–4, which examine the mechanism of action between Internet embeddedness and rural women’s nonfarm employment. In Table 3, model 1 tests Equation 2, model 2 tests Equation 3, and model 3 tests Equation 4.

^3^In this study, before starting the average treatment effect measurement, the matching results were tested for balance, and the results showed that the standardized bias value (bias) of each matched variable was less than 10% after matching, and the *p* values of the matched variables were significantly higher and none of the test results were significant, which means that the matching results were more accurate and reliable. For reasons of space, the matching results are not shown here, so please contact the author if you need.

^4^This study also used F test to verify the accuracy of the instrumental variables. The analysis showed a *p* value of 0, significantly rejecting the original hypothesis, which means that the instrumental variables selected for the article are accurate and reliable.
